# Supplementary material for: Human Skeletal Muscle Cells Derived from the Orbicularis Oculi Have Regenerative Capacity for Duchenne Muscular Dystrophy
Source: Int J Mol Sci. 2019 Jul 14;20(14):3456. doi: 10.3390/ijms20143456 (PMC6679213; doi:10.3390/ijms20143456)
Supplement: Supplementary file 1 [file ijms-20-03456-s001.pdf]

International Journal of *Molecular Sciences*

Article

Supplementary Material: Human Skeletal Muscle Cells Derived from the Orbicularis Oculi Have Regenerative Capacity for Duchenne Muscular Dystrophy

Yukito Yamanaka, Nana Takenaka, Hidetoshi Sakurai, Morio Ueno, Shigeru Kinoshita, Chie Sotozono and Takahiko Sato

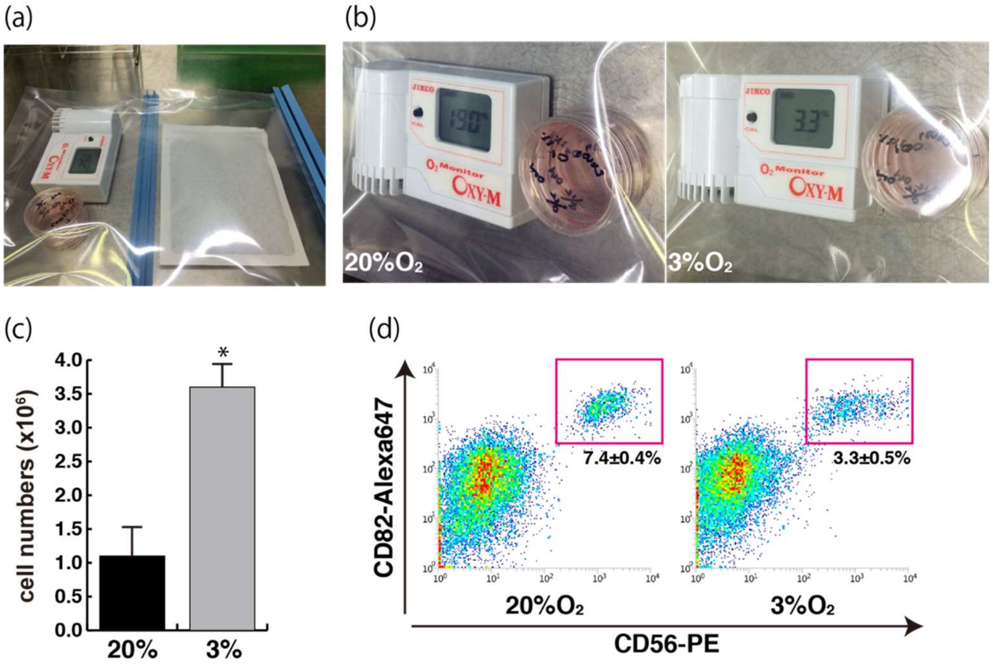

Figure S1. The hypoxia condition for myogenic cell culture with orbicular oculi. (**a**) For creating low oxygen tension for cell culture, the hypoxia incubation pack was prepared (BIONIX, Sugiyamagen). (**b**) The oxygen concentration of hypoxia incubation pack can be controlled from 20% (left panel) to 3% (right panel). (**c**) The total cell number of cultured CD56+cells controlled the oxygen concentration. All error bars indicate ±SEM ($n = 3$). P-values are determined by t-test from a two-tailed distribution. *$P < 0.01$. (**d**) FACS profile of cultured CD56+CD82+cells with or without the hypoxia condition.
